# Observations and Experiments on the Use of Calcined Magnesia as an Antidote to Arsenic

**Published:** 1846-10

**Authors:** Robert Peter

**Affiliations:** Professor of Chemistry and Pharmacy in the Medical Department of Transylvania University


2. *Observations and Experiments on the use of Calcined Magnesia as an Antidote to Arsenic.* By Robert Peter, M.D., Professor of Chemistry and Pharmacy in the Medical Department of Transylvania University.

Since the announcement, by MM. Bunsen and Berthold, in 1834, of the efficacy of the hydrated peroxide of iron as an antidote to the poisonous effects of arsenious acid, the attention of toxicologists has been so strongly fixed on the properties of this substance, that they have in a great measure lost sight of the means formerly relied on in this species of poisoning.

It is very true that many of them are worthless as *antidotes*, as has been proved to be the case with charcoal, vinegar, sugar, butter, limewater, bitter decoctions, &c.; and that one, the liver of sulphur, is itself a poison; but there seems to have existed no good reason why the virtues of magnesia, as an antidote to arsenic, should have remained so long unappreciated.

This is the more remarkable from the fact, that "Mr. Hume of London, and others, have apparently found advantage from the administration of large doses of magnesia,"* in cases of poisoning with arsenic. Yet Orfila makes no mention of this substance in his *Toxicologie*, (4th edition,) although he elaborately disproves the alleged efficacy of charcoal; and Christison, placing it in the same category with "the fine impalpable powders, inert as physiological agents, and destitute of any true chemical action with oxide of arsenic;" remarks: "If this substance be of any use at all, which is doubtful, it can only act by covering the arsenical particles with its fine

* London Med. and Phys. Jour., xlvi., 466, 545. Mr. Edwards, ibidem, xlix, 117. Mr. Buchanan, London Med. Repos., xlx, 288. (Quoted from Christison on Poisons.)

insoluble powder, and so preventing them from coming in contact with the surface of the stomach; for in its state of magnesia it has no chemical action with oxide of arsenic."—(*Christison on Poisons.* American edit. 1845. p. 283.)

Chemical antidotes are those substances which, by combining with the poisons, or decomposing them, either change their properties, and thus disarm them of their corrosive or irritant action, or form with them new compounds which are inert in consequence of their insolubility in the fluids of the stomach. Thus, magnesia will neutralize the acids by combining with them, and thus avert their corrosive action; or it will decompose certain metallic salts, and reduce them to an insoluble and comparatively harmless state; and it will also, as we think we can demonstrate, combine directly with arsenious acid to form *an arsenite of magnesia*, which is insoluble in the ordinary fluids of the stomach.

The reactions of magnesia with arsenious acid do not appear to have been much studied by chemists. It is true that the *arsenite of magnesia* is mentioned among the salts, and is stated to be *insoluble in water.* The neutral *arsenite* of magnesia, (the salt formed by the union of the arsenic acid with magnesia) is also said to be insoluble. Yet the most simple experiment would demonstrate the error of Dr. Christison in his statement that "magnesia has no chemical action with oxide of arsenic."

The attention of the writer was drawn to this subject by the perusal of the following summary of facts, submitted to the French Academy of Science, by M. Bussy, as the results of his labors, and published in the *Comptes Rendus.* 18 Mai, 1846. p. 845. viz:

"1. That purified animal carbon, recently proposed, cannot be successfully employed as an antidote to arsenic.

"2. That pure magnesia, which has not been intensely heated, absorbs arsenious acid in solution, and forms with it a compound which is insoluble even in boiling water.

"3. That in the gelatinous state it absorbs it still more promptly.

"4. That animals to which arsenic had been administered, were always saved when we caused them to take sufficient doses of magnesia.

"5. That this antidote presents the following advantages over those which are at present employed, viz: that it can always be obtained at the apothecary's shop, that it easily and completely neutralizes the poison, and may be administered in a large dose without inconvenience, its general therapeutical effects being in accordance with the indications in this kind of poisoning.

"6. That magnesia decomposes tartar emetic, the salts of copper, and corrosive sublimate;" and there is reason to believe that we may employ it with success to counteract the effects of those poisonous substances, as well as those of the metallic salts generally.

"7. That the salts of the organic alkalies, morphine, strychnine, &c., being also decomposed by magnesia, the administration of that substance in cases of poisoning with the organic products which owe their activity to the presence of the vegetable alkalies, might retard, and cause to be more difficult, the absorption of the poison,—this supposition it is proposed to verify by ulterior experiments."

Wishing to ascertain how far the results of M. Bussy, in relation to arsenic, could be verified, by the use of the ordinary calcined magnesia of the shops, the writer of the present article performed the following experiments.

In the first place, a solution was made by dissolving forty grains of pure lump white arsenic, reduced to powder, in five fluid ounces of distilled water, at a boiling temperature. The solution therefore contained one grain of arsenious acid to the fluid drachm.

*Experiment* 1.—One fluid drachm of the solution of arsenic was mixed with one drachm of calcined magnesia, and fifteen drachms of distilled water. The mixture was heated in a Florence flask up to about 150° Fah., when the lamp was removed, and it was allowed to stand about fifteen minutes. It was then filtered through paper and the clear solution tested for arsenic with *ammonio-nitrate of silver.* The delicate reagent only rendered the fluid slightly turbid, with a light colored, nearly white, precipitate. The addition of nitric acid, in which most of the light precipitate was insoluble, showed that only a *trace* of arsenite of silver was present, and that most of the precipitate was chloride of that metal, probably derived from the impurities of the magnesia. *Sulphuretted hydrogen* solution was then added to some of the filtered liquid previously acidulated with hydrochloric acid, and this test, which, according to Mohr, will indicate the presence of one part of arsenic in 200,000 parts of water, produced scarcely a perceptible change of color.

A current of sulphuretted hydrogen gas was then passed through the acidulated filtered liquid, for half an hour, which caused it to assume a scarcely perceptible yellow tinge, indicating the presence of a *mere trace* of the arsenious acid. Similar experiments with the above tests, made on a mixture of 1 fluid drachm of the original arsenic solution and fifteen drachms of water, without magnesia, gave well marked and copious precipitates in both cases.

The solution submitted to the action of the drachm of magnesia, contained one grain of arsenious acid in two ounces of water. The arsenic had evidently been almost entirely withdrawn from the solution by the magnesia; for th[illegible]

tests used,—one of which will indicate the presence of that poison in the minute proportion stated above, and the other, the ammonio-nitrate of silver, still more sensitive, is capable of showing an evident reaction with only one part of arsenic in 400,000 parts of water,—were both brought near to the extreme limits of their power by the dilute solution.

The magnesia remaining on the filter was then washed with cold water, after which it was subjected to the action of boiling water; to which it did not communicate a trace of arsenic discernible by the silver test.

*Experiment* 2.—A fluid drachm of the original arsenic solution was again mixed with fifteen drachms of distilled water and one drachm of calcined magnesia, and the mixture was allowed to remain, *without application of heat*, at the temperature of 77° Fah., for twenty minutes, when it was thrown upon a filter. The filtered fluid gave a *slight* precipitate with the ammonio-nitrate of silver, which only partially dissolved in nitric acid, but was wholly soluble in ammonia. Sulphuretted hydrogen produced only a tinge of yellow in the acidulated fluid, but caused no sensible precipitate.

Comparative experiments with a solution of the strength of that submitted to the action of the magnesia, showed clearly that the greater part of the arsenic had been removed from the liquid by that substance acting at the temperature of 77°. As it was desirable to know *how much* of the arsenious acid would be removed from the solution of that substance by a given quantity of magnesia, the following experiment was made.

*Experiment* 3.—One fluid ounce of the original arsenic solution, containing eight grains of that substance, was mixed with a fluid ounce of water and a drachm of the magnesia, and heated up to blood-heat. After allowing it to stand for ten minutes it was filtered, and half a fluid ounce of the filtered fluid was carefully evaporated to dryness in a weighed porcelain capsule. The fluid became turbid with a white precipitate on attaining the boiling point, and appeared of a pasty consistence when nearly evaporated. On re-weighing the capsule, it was found to have gained one grain and nine-tenths of a grain. The capsule containing the residuum was then strongly heated, to drive off the arsenious acid, and on weighing it again, the loss in weight was found to be one grain and one tenth; which was the amount of the arsenic contained in the half an ounce of the filtered solution. As the arsenic solution which was submitted to the action of the magnesia, in this experiment, contained two grains of arsenic to the half ounce, or eight grains of that substance in the whole mixture, it is evident that the drachm of magnesia had withdrawn four grains and four-tenths of the arsenious acid from the solution; a proportion nearly as great as that which would have been removed by hydrate of the peroxide of iron under the same circumstances, and which, from the manner of the performance of the experiment, does not give us the measure of the full extent of the action of magnesia on arsenic in solution.

It is evident from these facts, that magnesia would be a most useful agent in the treatment of poisoning with arsenic. Mixed with water, and administered continually, and in sufficient quantity, until all the poison is removed from the stomach, it would combine with the arsenious acid as soon as it became dissolved in the fluid of the stomach, and by withdrawing it immediately from solution, prevent its absorption into the living tissues. Even when the poison is taken in solution, we could doubtless succeed, by the speedy administration of a large quantity of this antidote, in gaining time to remove it from the stomach by the ordinary emetic means.

It is believed to be always desirable to evacuate the stomach freely after the use of *any* of the antidotes; more especially in poisoning with metallic substances; for, although the poison may for the time being, be converted into a compound insoluble in water, yet it might not be safe to allow that substance to remain long in contact with the secreted acids of the stomach.

Many persons may be disposed to think that, having in the hydrated peroxide of iron, a good antidote to arsenious acid, it would be folly to throw it aside for another which has not yet been sufficiently submitted to the severe test of experiment on the living subject. But the writer would suggest that the compound of iron is not always to be procured in time when it is wanted, and moreover is often inert from the improper manner of its preparation, while magnesia is almost always at hand, and can, generally, be procured in quantity; and it presents a range of antidotal power over various poisons not equalled by any other known substance. It is, therefore, to be strongly recommended as a substitute for the peroxide of iron, when the latter cannot be easily obtained.—*West. Lan.*

